# Insights into the Deep Phylogeny and Novel Divergence Time Estimation of Patellogastropoda from Complete Mitogenomes

**DOI:** 10.3390/genes13071273

**Published:** 2022-07-18

**Authors:** Jiantong Feng, Jing Miao, Yingying Ye, Jiji Li, Kaida Xu, Baoying Guo, Xiaojun Yan

**Affiliations:** 1 National Engineering Research Center for Marine Aquaculture, Zhejiang Ocean University, Zhoushan 316022, China; fengjiantong0109@163.com (J.F.); z20095136252@zjou.edu.cn (J.M.); lijiji@zjou.edu.cn (J.L.); guobaoying@zjou.edu.cn (B.G.); 2 National Engineering Laboratory of Marine Germplasm Resources Exploration and Utilization, Zhejiang Ocean University, Zhoushan 316022, China; 3 Marine Fishery Institute of Zhejiang Province, Key Laboratory of Sustainable Utilization of Technology Research for Fishery Resource of Zhejiang Province, Zhejiang Ocean University, Zhoushan 316021, China; xkd1981@163.com

**Keywords:** mitochondrial genome, Patellogastropoda, *Cellana toreuma*, gene order, phylogeny, divergence time

## Abstract

To further understand the origin and evolution of Patellogastropoda, we determined the mitochondrial genome sequence of *Cellana toreuma*, and compared its mitogenome characteristics with the other four limpets of Nacellidae. The ratio of Ka and Ks indicated that these Nacellidae species were suffering a purifying selection, with exception of the *atp6* gene. The gene sequence is basically consistent among families, while there are great differences among Lottidae species. According to the mitogenome sequences of selected gastropod species, we reconstructed a new phylogenetic tree with two methods. The data complement the mitogenome database of limpets and is a favorable research tool for the phylogenetic analysis of Gastropoda. It is found that there is a long-branch attraction (LBA) artefact in the family Lottiidae of Patellogastropoda. Therefore, the Patellogastropoda was separated by Heterobranchia, and Lottiidae is located at the root of the whole phylogenetic tree. Furthermore, we constructed the divergence time tree according to the Bayesian method and discussed the internal historical dynamics, and divergence differences among the main lineages of 12 Patellogastropoda under an uncorrelated relaxed molecular clock. In turn, we made a more comprehensive discussion on the divergence time of limpets at the molecular level.

## 1. Introduction

Mitochondria are circular double-membrane semiautonomous organelle, which exist in the cells of most eukaryotic species. It has an independent and complete mitochondrial genome. They originate from an endosymbiotic α-proteobacterium and usually provide chemical energy sources through oxidative phosphorylation [1,2,3,4]. Because mutations affecting mitochondrial function are related to aging and disease, it also has certain biomedical significance [5,6]. The advantages of mitochondria are that their evolution rate is faster than that of nuclear genes in most species. Each cell has multiple copies of the mitochondrial genome and higher A-T content [7]. Moreover, it also has the characteristics of conservation gene function [8]. Mitogenome recombination is a common process in protists and plants [9,10]. Single genes may affect the progress of species relationships because of their different evolutionary rates [11]. Thus, the complete mitogenome is considered significant in population genetics and phylogeny, as well as an important tool for an in-depth understanding of gastropod phylogenetics.

Patellogastropoda, as an archaic mollusk, has caused concern in the scientific community. Due to their important ecological status and biodiversity, it is often researched in morphology, ecology, biogeography, embryology, and population genetics [12,13,14,15,16,17,18]. Patellogastropoda has assumed a significant role in the evolution of marine organisms among the gastropods on intertidal rocky shores. They have experienced a long period of species evolution, while the current classification indicates that Patellogastropoda includes the two superfamilies of Lottioidea (Gray, 1840) and Patelloidea (Rafinesque, 1815) [19]. According to the latest classification, the superfamily Lottioidea includes seven families (Acmaeidae, Eoacmaeidae, Erginidae, Lepetidae, Lottiidae, Neolepetopsidae, and Pectinodontidae), while the superfamily Patelloidea has only two families (Nacellidae and Patellidae) [19]. Previous identification of Patellogastropoda species was based on morphology and has caused taxonomic confusion. Limpets are considered cryptic species and the appearance of different species is almost similar. Surprisingly, the immature forms of limpets differ from the adults, which causes more complications for species identification.

The small economic limpet *Cellana toreuma* (Reeve, 1854) is found on sheltered to intertidal rocky shores and is widely distributed from tropical to Polar regions [20] this includes mainly China, Japan, South Korea, Ryukyu, Vietnam, and the Philippines [21,22,23,24,25,26,27]. The limpet is an important grazer and an ectoparasitic host of *Philoblenna tumida* (Ho, 1981), Hexanauplia [28,29,30]. Due to the complex biogeographic model of limpets, they are used to understand the distribution pattern of species in the intertidal zone along heterogeneous coastline [31,32,33,34]. Their shell is a rare Chinese herbal medicine, which is often collected and processed in summer [35]. It is mainly used for children with convulsions and other symptoms to implement a sedative effect [36]. The duration of planktonic larvae of *C. toreuma* is uncertain, while the reported results show that they can last for at least 8 to 13 days [37,38]. *C. toreuma* is sensitive to ambient temperature, and high temperature will affect cardiac performance and lead to large-scale mortality [39,40,41]. Early reports mention studies and analyses being conducted on morphological and population dynamics. Firth and Williams [37] used multiple environmental stressor influences to study the population dynamics of *C. toreum*. The effects of large changes in temperature and salinity associated with wet monsoon seasons on the structure and function of tropical rocky coasts are thus revealed. Wang and Wu [42] collected and measured data from northern Zhejiang, China, and studied the age and growth of *C. toreum*. It is concluded that their annual ring formation period is in January and the cycle is one year. Qian et al. [43] reported optical and electron microscopy to observe the radular morphological differences between *C. toreuma* and *Cellana grata*. Hirano [44] mentioned the activity pattern of *C. toreuma* field population and learned that it is more frequent at flood and ebb tide. Iwasaki [45] studied the interindividual trail following *C. toreuma* and found that they moved farther during spring tides. However, there have only been a few reports on the phylogenetic studies of this species. Wang et al. [24] selected a single mitochondrial COI to reveal the phylogeographical pattern of *C. toreuma* and investigate the effect of environmental and historical factors on this pattern. Similarly, Nakano [27] discovered a hidden species of *C. toreuma* through the COI gene and analyzed its fundamental evolutionary relationship. Until the 21st century, Nakano and Ozawa [26] presented a comprehensive phylogenetic study on the global limpets and combined mitochondrial and nuclear sequencing. These are just the preliminary study results of Patellogastropoda phylogeny.

*Cellana toreuma*, as one of the few mitochondrial whole-genome sequences of Patellogastropoda, provides a further supplement for the phylogenetic analysis of Patellogastropoda. Our aims were to (1) extend the taxonomic study method and improve the Patellogastropoda identification efficiency, (2) compare and evaluate the variation and conservation of mitogenomes to understand the latest classification of Patellogastropoda, (3) via A-T skew values and the relative synonymous codon usage (RSCU) of protein-coding genes (PCGs), understand the gene function, (4) establish a complete analysis system for gastropod phylogeny, especially for limpets evolution, and (5) evaluate divergence time of *C. toreuma* in subclass Patellogastropoda given the fossil record, so as to study the evolutionary history of limpets.

## 2. Materials and Method

### 2.1. Sample Collection, Identification, and DNA Extraction

*Cellana toreuma* wild specimens were collected from Qingdao of Shandong Peninsula in the Yellow Sea (October 2019; E 120° 45, N 36° 07). The specimens were deposited in absolute ethyl alcohol. The specimens were preliminarily identified through the published taxonomic book [46], and we consulted morphology experts from the marine biology museum of Zhejiang Ocean University. Fresh samples were immediately placed in absolute ethyl alcohol to ensure their quality. Using the rapid salting-out method, we extracted the genomic DNA from the adductor muscle [47]. The quality was determined by 1% agarose gel electrophoresis and stored in −20 °C for sequencing. We selected the best quality DNA from the six samples for the next-generation sequencing. All animal experiments were conducted under the guidance approved by the Animal Research and Ethics Committee of Zhejiang Ocean University.

### 2.2. Mitogenomes Sequencing, Assembly, and Annotation

Mitogenome sequencing of *C. toreuma* by the Illumina HiSeq X Ten platform was used to conduct high-throughput sequencing; this work was carried out by Origingene Bio-pharm Technology Co., Ltd. (Shanghai, China). The preliminary results showed that a sequencing library set with an average insert size of 400 bp was generated, and each library had about 10 Gb of the raw data. After that, it was necessary to delete contaminated reads and low-quality sequence fragments. The de novo assembled separate clean readings of the sequence via the NOVOPlasty software (https://github.com/ndierckx/NOVOPlasty (accessed on 26 May 2021)) [48].

The mitochondrial genome of *C. toreuma* was annotated and analyzed based on invertebrate genetic code by the MITOS web server (http://mitos2.bioinf.uni-leipzig.de/index.py (accessed on 30 May 2021)) [49]. We also referred to the uploaded mitogenome sequence of other Nacellidae species to ensure the accuracy of start and stop codons and gene sequences of the species in our study. The circular mitogenome visualization of *C. toreuma* was completed through the common CGView server (http://stothard.afns.ualberta.ca/cgview_server/index.html (accessed on 30 May 2021)) [50].

### 2.3. Sequence Analyses of Mitogenomes

The nucleotide composition of the whole mitogenome, PCGs, rRNA, tRNA genes, and A-T content were analyzed by MEGA 7.0 [51]. Meanwhile, it was also determined necessary to study the codon usage and the relative synonymous codon usage (RSCU) of PCGs. Then, the base skew values were calculated using the formulas at A-T skew = (A − T)/(A + T) and G-C skew = (G − C)/(G + C) [52]. In addition, we selected DnaSP6.0 [53] to analyze the non-synonymous (Ka) and synonymous (Ks) substitutions rates of mitogenomes in Nacellidae species to study their evolutionary adaptation. Of which other species in Nacellidae were downloaded from the GenBank database of NCBI (National Center for Biotechnology Information, https://www.ncbi.nlm.nih.gov/ (accessed on 17 March 2022)).

### 2.4. Phylogenetic Inference

To determine the phylogenetic position of the Patellogastropoda species in gastropods, phylogenetic analyses were performed based on the 13 protein-coding genes (PCGs) of the mitogenomes. A total of 87 sequences were downloaded from GenBank (https://www.ncbi.nlm.nih.gov/ (accessed on 17 March 2022)). Furthermore, two bivalves *Donax variegatus* and *Donax trunculus* were classified as outgroup [54] (Table 1). The software DAMBE 5.3.19 [55] was used to adjust the nucleotide sequence of each PCGs, and the substitution saturation was calculated via the GTR substitution model. The sequences were aligned using ClustalW of MEGA 7.0 [51] with the default parameters. Phylogenetic relationships were reconstructed for the maximum likelihood (ML) and Bayesian inference (BI) analyses with IQ-TREE [56] and MrBayes v3.2 [57]. The GTR + F + I + G4 model was chosen according to BIC and was the best fit selected in the ML methods analyses; we used ModelFinder to determine the best model data [58]. We reconstructed the consensus tree and used 1000 bootstrap replicates in ultrafast likelihood bootstrap. The BI analysis selected the best substitution GTR + I + G model under the AIC by MrModeltest 2.3 [59]. The first burn-in 25% of the trees were discarded, and two Markov chain Monte Carlo (MCMC) of simultaneous were operated for 2,000,000 generations. Sampling occurred every 1000 ultrafast bootstrap replicates to determine the branch support of the dataset. The phylogenetic tree was displayed using the online tool iTOL (Interactive Tree of Life, https://itol.embl.de/ (accessed on 20 March 2022)) and annotated with various datasets [60].

### 2.5. Divergence Time Estimation

The divergence time of the subclass Patellogastropoda species was estimated only at the nucleotide level, 13 PCGs used the Bayesian framework, and we used the uncorrelated and lognormal relaxed molecular clock model in BEAST v1.8.4 [61]. For the tree prior, we used a Yule process of speciation. Furthermore, we used two calibration points as the priors of the divergence times for calibration. The uniform distribution of the estimated divergence times was drawn by Priors [62] for fossil ages, and the 53 Mya point calibration was set as the root rate of *Cellana* based on the fossil of *Cellana tramoserica* (14–93 Mya). The 5.6 Mya point calibration was set as the root rate of *Nacella* based on the fossil of *Nacella clypeater* [62]. The final Markov chain samples every 1000 generations, discards 10% of the burn-in samples, and runs twice for 100 million generations through the TreeAnnotator v1.8.4 software in the BEAST software package. After that, we use Tracer v. 1.6 [63] to check the convergence of the chain, to ensure that the parameters of the effective sample sizes (ESSs) were greater than 200. We used the software FigTree v1.4.3 to visualize the divergence time tree [64].

## 3. Results and Discussion

### 3.1. General Features of Entire Mitogenome

**Figure 1:**
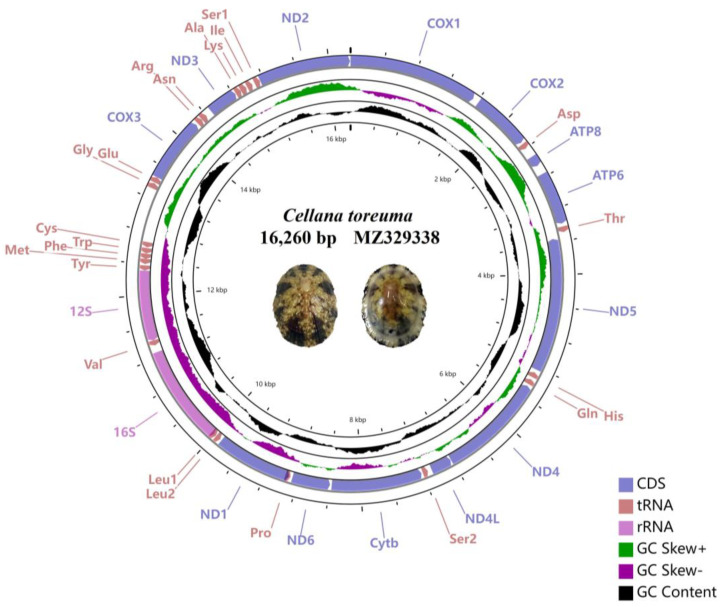
Gene map of the complete mitogenomes for *Cellana toreuma* (GenBank accession No. MZ329338). The ring indicates gene arrangement and distribution.

The whole mitogenome sequence of *C. toreuma* was sequenced with a length of 16,260 bp (GenBank accessions: MZ329338) which is consistent with previously reported species of the four Nacellidae families, approximately 16,153 to 16,767 (Table 1). The circular molecules are similar to other gastropods, which contain a highly variable control region and typically 37 genes including 2 ribosomal RNA genes, 13 protein-coding genes (PCGs), and 22 transfer RNA genes. Among them, a total of seventeen genes on the forward strand, including seven PCGs (*cox1-3*, *atp8*, *atp6*, *nad3*, and *nad2*), and ten tRNA genes (*trnD*, *trnT*, *trnG*, *trnE*, *trnR*, *trnN*, *trnA*, *trnK*, *trnI*, and *trnS1*). The other genes are encoded on the reverse strand (Table 2). The control region was located between the *trnC* and *trnG* gene, similar to other previously reported Nacellidae species (Figure 1) [65,66]. The genome structure of *C. toreuma* was identical to other Nacellidae mitogenomes, without gene rearrangement in this family. However, there was a big difference between their gene order to other families of Patellogastropoda species, whose rearrangement always brought up concern within the scientific community.

The nucleotide compositions of complete *C. toreuma* mitogenomes were A 28.9%, T 39.5%, G 19.9%, and C 11.7% (Table 3). Moreover, the nucleotide compositions of the mitogenomes from the other four species in the family Nacellidae, Patellogastropoda were downloaded and organized, and we compared the base compositions of these other Nacellidae species. In general, the A content of the five mitogenomes were from 26.5 to 28.9%, T 38.0 to 39.5%, G 19.9 to 22.7%, and C 11.7 to 13.9%, these base contacts only had a slight distinction. Results show that the A and T content of *C. toreuma* exhibited higher values than other species in the same family, with a range from 64.6% (*Cellana nigrolineata*) to 68.4%. The nucleotide compositions were all skewed away from C in favor of G, the G-C skews were from 0.177 (*N. clypeater*) to 0.283 (*C. nigrolineata*), and from A in favor of T, the A-T skews were negative from −0.180 (*Nacella concinna*) to −0.155 (*C. toreuma*), indicating the occurrence of more Ts than As.

### 3.2. tRNA, rRNA, PCGs Genes, and Control Region

For the tRNA genes of *C. toreuma*, the length ranged from 1497–1560 bp, which had an A and T content of 69.6%, which is the highest compared to other Nacellidae species (Table 3). Due to the tRNAs of this limpet having similar values of A and T base, the A-T skew was 0, while the A-T skew of the other four species were negative. However, all the G-C skews were slightly positive from 0.103 (*C. toreuma*) to 0.125 (*N. clypeater*). For the rRNA genes, with lengths ranging from 2143 to 2222 bp, the A-T skews were positive from 0.169 (*C. toreuma*) to 0.237 (*N. concinna*), indicating a strong skew away from A. Furthermore, almost all G-C skews of these species were negative, except the new limpet *C. toreuma*.

For the PCGs, the length ranging from 11,046 to 11,286 bp, each species in this family exhibited a negative A-T skew and a positive G-C skew, with values from −0.221 to −0.201. The first codon position of PCGs was observed to be a negative A-T skew, with the A-T content reaching about 65%, and the most value being 67% in *C. toreuma*. The second and third codon positions of PCGs were similar, while their G-C skews had the opposite results. The mitogenomes of Nacellidae are rich in A-T, which is similar to other invertebrates. The *C. toreuma* mitogenomes conventional started with the initiation codon ATG or ATT and stopped with TAA or TAG.

Generally, the mitogenome of metazoans is quite compact, whereas a total of 1641 bp in 30 intergenic spacers were found in *C. toreuma* mitogenome, ranging from 1 to 643 bp in length. Additionally, the control region was found between *trnC* and *trnG* (Table 2). Simultaneously, three overlapping sites (totally 87 bp) are observed ranging from 2 to 47 bp, which is commonly identified in other Nacellidae.

### 3.3. Mitochondrial Gene Codon Usage

**Figure 2:**
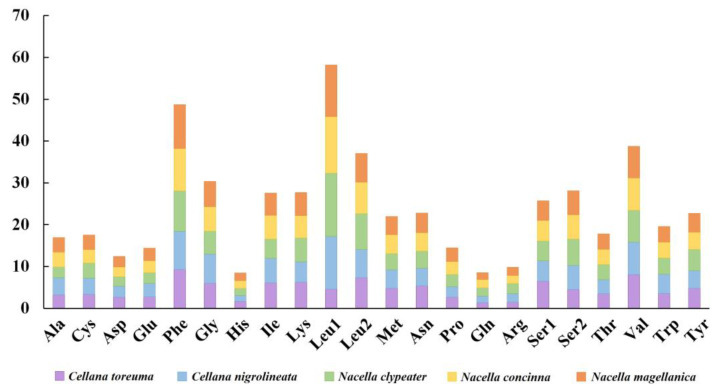
Percentage of each amino acid for proteins coded by PCGs in the five mitochondrial genomes of Nacellidae.

The amino acids of five Nacellidae species, Leu1, Phe, and Val, were most frequently utilized ranging from 7.60 to 15.11% in Phe of *N. clypeater* (Figure 2). The rare amino acid was concentrated in His, Gln, and Arg, most of them less than 2 with the least being Gln of *C. toreuma* (only 1.38%), which is similar to other Patellogastropoda as previously reported [67,68].

**Figure 3:**
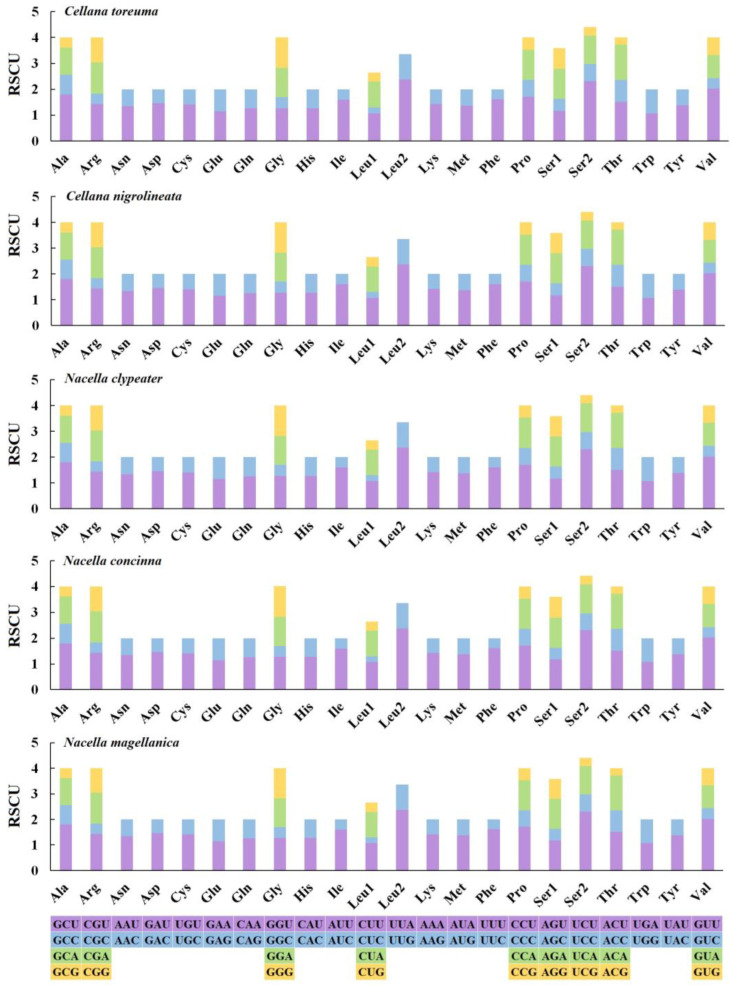
The relative synonymous codon usage (RSCU) in the mitochondrial genomes of five Nacellidae species.

The relative synonymous codon usage (RSCU) for 13 PCGs of these species was measured to understand the genetic codon bias of their sequenced mitogenomes. The results showed that the synonymous codon preferences are conserved among the five species, as may ascribe to their close relationships belonging to the same family. These preferences were also recognized in some other Patellogastropoda. The four most used codons for the five species sequenced are consistently UUA (Leu2), UCU (Ser2), GCU (Ala), and CCU (Pro) for five Nacellidae species (Figure 3), and the most frequent codons of them were UUA at 2.4% (Leu2) in *N. magellanica*. The least used codon was CUC (Leu1) in *C. toreuma,* which was 0.1%.

### 3.4. Selective Pressure Analysis

**Figure 4:**
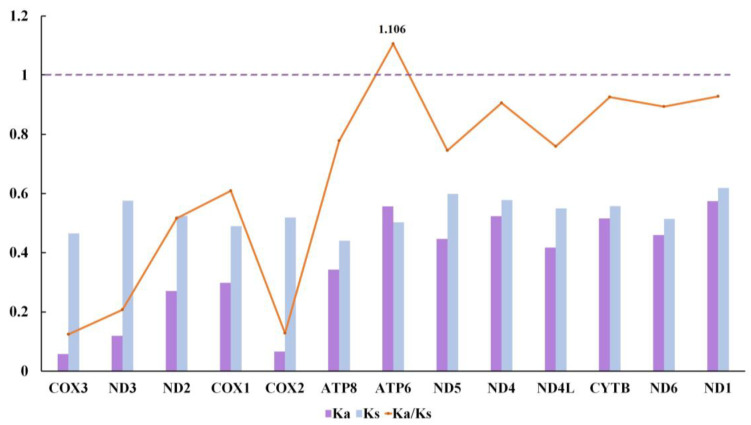
Selective pressure analysis for 13 PCGs among 5 Nacellidae mitochondrial genomes. Species of Nacellidae are shown in Table 1. The purple and blue boxes indicate the number of nonsynonymous substitutions per nonsynonymous sites (Ka) and the number of synonymous substitutions per synonymous sites (Ks), respectively. The orange line indicates the mean of pairwise divergence of the Ka/Ks ratio.

The selection pressure analysis also used these five species (Figure 4) to measure the ratio of non-synonymous and synonymous substitutions (Ka/Ks). We aimed to investigate the evolutionary and selective pressure relation. The results showed the average Ka/Ks ranging from *cox3* (0.124) to *atp6* (1.106). The ratio for most PCGs was below one, indicating that the mutations were swapped by synonymous substitutions; 13 PCGs of these mitogenomes were evolving under purifying selection. The remaining *atp6* gene reached 1.106, which may be due to the influence of positive selection during evolution. Among these species, the *cox3* gene had little change in amino acids and the lowest Ka/Ks ratio. It is widely used as a potential molecular marker for species identification, genetic diversity, and phylogenetic analysis [69,70].

The substitution saturation index of the combined dataset for 88 Patellogastropoda mitogenomes for 13 PCGs (Iss = 0.823) was significantly lower than the critical values (Iss.cSym = 0.860 or Iss.cAsym = 0.847, *p* = 0.000). Therefore, substitution of combined sequencing was unsaturated and suitable for phylogenetic analysis.

### 3.5. Gene Arrangement

**Figure 5:**
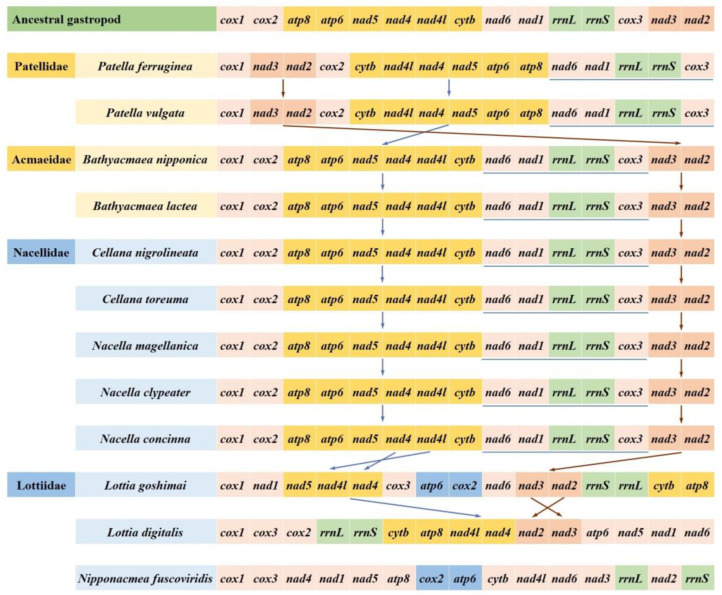
Comparison of mitochondrial gene order of the family Nacellidae in Patellogastropoda.

According to the hypothetical gene order of ancestral gastropods, we compared the PCG gene arrangement of 12 species in four families of the subclass Patellogastropoda (Figure 5). The results showed that the gene order of the family Nacellidae, where *C. toreuma* was located, was the same as that of the family Acmaeidae, and was consistent with the ancestral gene order. In Patellidae, we found that only the *nad3*-*nad2* fragment moved, and the position was transferred from one fragment of *cox1* to the other. The *nad6*-*nad1*-*rrnL*-*rrnS*-*cox3* fragment is still completely preserved. In addition, the *atp8*-*atp6*-*nad5*-*nad4*-*nad4l*-*cytb* gene fragment was completely reversed. It is worth noting that the gene rearrangement rate of the family Lottiidae is very high. Among them, *L. goshimai* retains the *nad3*-*nad2* gene fragment, while the *nad4* and *nad4l* genes in *L. goshima* are reversed; this situation also occurs in *Lottia digitalis*. Moreover, the short gene fragment of *rrnL*-*rrnS* was retained in *L. digitalis*, and two *rrnL* fragments were reversed in *L. goshimai*. In particular, *N. fuscoviridis* hardly retains the gene fragments of its ancestors; however, we can find that it has the same fragment *cox1*-*cox3* as *L. digitalis* in the same family. It also has the inversion of *atp6* and *cox2* genes with *L. goshima*, which may be the reservation of unique gene fragments produced in the evolutionary process of this family. In general, the rearrangement of Lottiidae remains the focus of our research. This irregular rearrangement may be responsible for the separation of this family from the other three families in the subclass Patellogastropoda.

### 3.6. Phylogenetic Relationship

**Figure 6:**
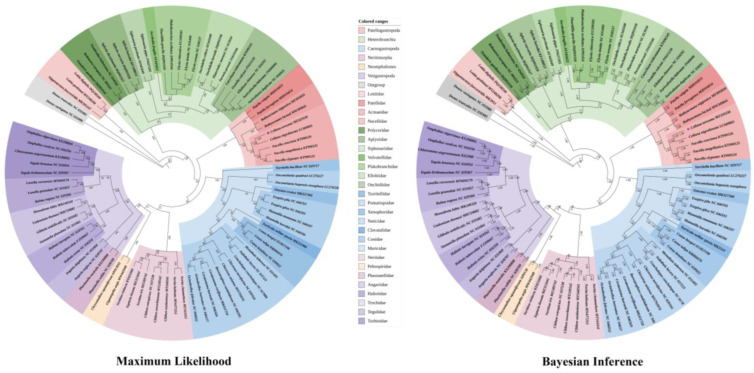
Phylogenetic tree inferred using maximum likelihood (ML) and Bayesian inference (BI) methods based on concatenated sequences of 13 PCGs from 88 gastropod mitogenomes. The sequences of two bivalves were chosen as the outgroups. The purple dots indicate *C. toreuma* sequenced in this study. The number at each node is the bootstrap probability.

After a long period of evolution, the species of Patellogastropoda are mainly divided into two superfamilies, Lottioidea (Gray, 1840) and Patelloidea (Rafinesque, 1815). We concatenated the alignment of 13 common PCGs from *C. toreuma*, as combined in 86 species that represent 26 families of these 2 superfamilies in Gastropoda, (i.e., Patellogastropoda, Heterobranchia, Caenogastropoda, Neritimorpha, Neomphaliones, and Vetigastropoda). The bivalves *D. variegatus* and *D. trunculus* were set as outgroups (Figure 6). For ML and BI trees, the 88 species could be divided into seven major clades. Of which most branches exhibited high confidence of being coincidently classified with different clades, (i.e., BI: 1 posterior probability and 100% bootstraps value). Strikingly, we found that the Patellogastropoda species were divided into two branches, located on both sides of Heterobranchia. The family Lottiidae of Patellogastropoda was located at the foundational position of the integral phylogenomic tree, while other families such as Patellidae, Acmaeidae, and Nacellidae were located between Heterobranchia and Caenogastropoda. We speculated that this is probably a result of the long-branch attraction (LBA) artefact. This phenomenon was also discovered in the study of two limpets *N. fuscoviridis* and *L. goshimai* [68]. The LBA artifact was defined as taxa with long branches that evolve rapidly in phylogenetic analysis, regardless of their real evolutionary location, which is highly misleading because the inferred false relationship is often highly statistically supported [71]. Both BI and ML analyses are generally unable to avoid this artifact due to the limitations of their model, and there is no good solution at present, which is also a place that needs to be improved for the future. Some scholars have studied and discussed the LBA artifact in previous reports, but no studies can counteract phylogenetics. We still obtain such a branch under our repeated verification in both methods. After investigating, we speculated that the LBA artifact often produces misleading results usually resulting from uneven or adequate sampling, and the mitogenome data of Patellogastropoda is insufficient and cannot be solved temporarily. This makes us more interested in the evolutionary research of the subclass Patellogastropoda, and it is imperative to continue to find new data on Patellogastropoda. After that, these clades combined at the subclass Caenogastropoda. Phylogenetic relationships of the subclasses are recovering as ((((((Vetigastropoda + Neomphaliones) + Neritimorpha) + Caenogastroopoda) + Patellogastropoda) + Heterobranchia) + Patellogastropoda). Their overall branching is basically consistent with our previous evolutionary analysis. Arquez et al. [72] used mitogenome to analyze the evolution of five subclasses of Gastropoda. The results showed that Heterobranchia and Patellogastropoda were sister groups to each other, and this branch was located at the root of the entire evolutionary tree. Caenogastropoda, Vetigastropoda, and Vetigastropoda had the same branch as our study [72]. Subsequently, Osca et al. [73] also used the mitogenome to study the phylogeny of Gastropoda species. However, their result was slightly different, Caenogastropoda and Neritimorpha were sister groups, followed by the subclass Vetigastropoda. In addition, Uribe et al. [74] obtained a similar result to Osca et al. [73]; nevertheless, they added the species of the subclass Neomphalina, which are situated between Vetigastropoda and the sister group Heterobranchia and Patellogastropoda. Sun et al. [75] reconstructed the tree of several species of gastropods using mitogenome, species from Cocculiniformia and Neomphalina have been updated and divided into the Neomphalines subclass. These results were completely consistent with the evolutionary branches of several subclasses in our study [75]. In addition, branches from the present study were similar to the results of Feng et al. [76], except for the minimal change of Caenogastropoda and Neritimorpha. The simple phylogenetic analysis of the deep-sea limpet by Li et al. [77] was the same as the results of the present study, which confirms the reliability of our predicted branches of the phylogenetic tree.

Patellogastropoda has four families that make up the whole mitogenome, Nacellidae and Acmaeidae are sister groups, followed by Patellidae. The Lottiidae, as an independent branch, is located at the outermost of this subclass. Furthermore, our research species *C. toreuma* and *C. nigrolineata* are undoubtedly sister species, both belonging to the genus *Cellana*. They were divided into one branch with three species of the genus *Nacella* in the same Nacellidae family. Phylogenetic trees of the Nacellidae families stand for (*C. toreuma* + *C. nigrolineata*) + (*N. concinna* + (*Nacella magellanica* + *N. clypeater*). These evolutionary branches conform to the conventional evolutionary characteristics, complying with the results of existing studies.

### 3.7. Divergence Times

We found that only seven available species of complete Patellogastropoda mitogenomes were recorded in the Timetree (http://www.timetree.org/ (accessed on 21 March 2022)) with only three known divergent time points. These were concentrated in *Nacella* and *Cellana* during the Cenozoic Era (65 million years ago, Mya) [78]. However, the divergence time information of *Bathyacmaea nipponica*, *Tectura paleacea*, and *Patella aspera* were unknown, which is a very unusual situation in Gastropoda species. Our study was designed to estimate more information on the evolutionary time and understand the historical evolution and dynamics of the limpets.

**Figure 7:**
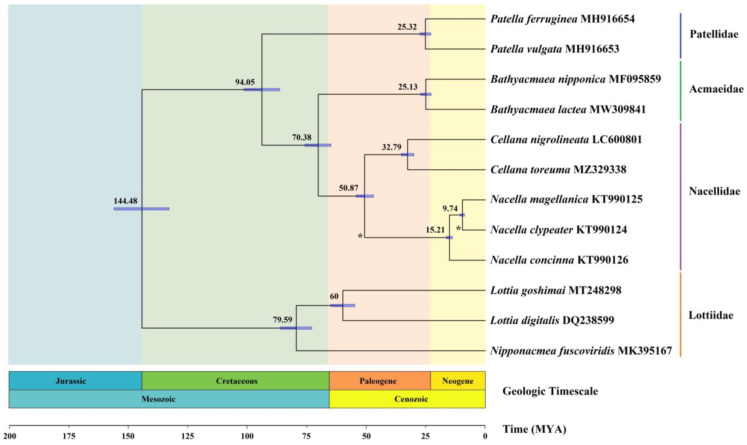
Divergence time estimation analysis of Nacellidae inferred via Bayesian relaxed dating methods (BEAST) based on the nucleotide sequences of 13 PCGs. Fossil samples used to calibrate internal nodes are represented by an asterisk. A total of 95% HPD is reported as blue bars, and Bayesian posterior probabilities are reported for each node.

Our study demonstrated that *N. magellanica* and *N. clypeater* were differentiated around 9.74 Mya, and *N. concinna* differentiated at 15.21 Mya (Figure 7). They are slightly earlier than the results in Timetree, but all the current differentiation occurred in the Neogene era. *Nacella* and *Cellana* were differentiated around 50.87 Mya, which is consistent with previous estimates of the divergence time. The analyses supported the supposition that the major Patellogastropoda lineages originated in the early Cretaceous and diversified in the middle and later Cretaceous; these have never been reported previously. In addition, the *N. fuscoviridis* family diversified to the genus *Lottia* in the later Cretaceous. The genera *Lottia*, *Bathyacmaea*, *Patella*, and *Cellana* were differentiated into Cenozoic Paleogene. All Nacellidae species differentiation is concentrated in the Cenozoic period. The geographical isolation in this period provided the environmental conditions for the Nacellidae differentiation, and marine sediments provided food sources for the growth of Patellogastropoda.

## 4. Conclusions

To carry out a good systematic study of limpets, a new potential species was found, and the mitogenomes of two species in the family Lottiidae were published. This provided more insights into mitogenomes in Patellogastropoda and supplemented the molecular database in such a classification group. We obtained the mitogenome sequences of *C. toreuma* by high-throughput sequencing with the length of 16,260 bp, similar to other limpets. Each mitogenome has the same composition and similar results of nucleotide composition for five Nacellidae. The gene order was generally uniform within families, except for the family Lottidae. Most PCGs were initiated with the ATG codon and terminated with TAA codon. For the analysis of selective pressure, we found that most of the 13 PCGs of Nacellidae were below 1, especially the *cox3* gene which exhibited the lowest value that demonstrated high conservation. This indicates that PCGs were subject to purifying selection in the family, while the *atp6* gene shows a high value, indicating that this gene may have been mutated in the process of evolution. The phylogenetic tree provided a further complement to the scientific classification of Patellogastropoda species. It is found that there is an LBA artifact in the family Lottiidae in Patellogastropoda, which deceived phylogenetic methods caused by the outgroup as distant or that the taxon sampling is poor. To solve this problem, we use two methods to construct evolutionary trees, but the Patellogastropoda is also divided into two branches on both sides of Heterobranchia. This study could provide the basic information for genetic characteristics, phylogenetic position, and evolution for these limpets, and provide a basis for resource management and selective breeding in aquaculture. This *Cellana* species was differentiated in the late Paleogene, and their evolution may be related to the geological events that changed their living environment.

## Figures and Tables

**Table 1:** List of species analyzed in this study and their GenBank accession numbers.

Subclass	Family	Species	Size (bp)	Accession No.
Patellogastropoda	Lottiidae	*Nipponacmea fuscoviridis*	18,720	MK395167
		*Lottia goshimai*	18,192	MT248298
		*Lottia digitalis*	26,835	DQ238599
	Patellidae	*Patella vulgata*	14,808	MH916653
		*Patella ferruginea*	14,400	MH916654
	Acmaeidae	*Bathyacmaea nipponica*	16,792	MF095859
		*Bathyacmaea lactea*	18,446	MW309841
	Nacellidae	***Cellana toreuma***	**16,260**	**MZ329338**
		*Cellana nigrolineata*	16,153	LC600801
		*Nacella concinna*	16,761	KT990126
		*Nacella magellanica*	16,663	KT990125
		*Nacella clypeater*	16,742	KT990124
Heterobranchia	Polyceridae	*Notodoris gardineri*	14,424	DQ991934
		*Roboastra europaea*	14,472	NC_004321
		*Nembrotha kubaryana*	14,395	NC_034920
	Aplysiidae	*Aplysia dactylomela*	14,128	DQ991927
		*Aplysia vaccaria*	14,130	DQ991928
		*Aplysia kurodai*	14,131	KF148053
	Siphonariidae	*Siphonaria pectinate*	14,065	AY345049
		*Siphonaria gigas*	14,518	JN627205
	Volvatellidae	*Ascobulla fragilis*	14,745	AY345022
	Placobranchidae	*Thuridilla gracilis*	14,259	DQ991939
		*Plakobranchus ocellatus*	14,173	AP014544
		*Elysia chlorotica*	14,132	EU599581
		*Elysia timida*	14,088	NC_035490
		*Elysia ornata*	14,188	NC_030537
	Onchidiidae	*Onchidella celtica*	14,150	AY345048
		*Onchidella borealis*	14,510	DQ991936
		*Platevindex mortoni*	13,991	NC_013934
		*Peronia peronii*	13,968	JN619346
	Ellobiidae	*Carychium tridentatum*	13,908	KT696545
		*Ovatella vulcani*	14,274	JN615139
		*Ellobium chinense*	13,979	NC_034292
		*Auriculinella bidentata*	14,135	JN606066
		*Auriculastra duplicata*	13,920	NC_036959
Caenogastropoda	Turritellidae	*Turritella bacillum*	15,868	NC_029717
	Pomatiopsidae	*Oncomelania quadrasi*	15,184	LC276227
		*Oncomelania hupensis nosophora*	15,182	LC276226
	Xenophoridae	*Onustus exutus*	16,043	MK327366
	Naticidae	*Euspira pila*	15,244	NC_046703
		*Euspira gilva*	15,315	NC_046593
		*Mammilla mammata*	15,319	NC_046597
		*Mammilla kurodai*	15,309	NC_046596
	Turridae	*Turricula nelliae spuria*	16,453	MK251986
	Conidae	*Conus borgesi*	15,536	EU827198
		*Conus tulipa*	15,756	KR006970
		*Conus betulinus*	16,240	NC_039922
	Muricidae	*Menathais tuberosa*	15,294	NC_031405
		*Indothais lacera*	15,272	NC_037221
		*Concholepas concholepas*	15,495	NC_017886
		*Chicoreus torrefactus*	15,359	NC_039164
		*Chicoreus asianus*	15,361	MN793976
		*Boreotrophon candelabrum*	15,265	NC_046505
		*Ceratostoma rorifluum*	15,338	MK411750
		*Ceratostoma burnetti*	15,334	NC_046569
		*Ocinebrellus inornatus*	15,324	NC_046577
		*Ocinebrellus falcatus*	15,326	NC_046052
Neritimorpha	Neritidae	*Nerita chamaeleon*	15,716	MT161611
		*Nerita balteata*	15,571	MN477253
		*Clithon oualaniense*	15,706	MT568501
		*Clithon sowerbianum*	15,919	MT230542
		*Clithon retropictus*	15,802	NC_037238
		*Neritina iris*	15,618	MW694828
		*Septaria lineata*	15,697	MW694829
		*Neritina violacea*	15,710	KY021066
Neomphaliones	Peltospiridae	*Chrysomallon squamiferum*	15,388	AP013032
		*Gigantopelta aegis*	16,097	MW442948
Vetigastropoda	Phasianellidae	*Phasianella solida*	16,698	NC_028709
		*Phasianella australis*	18,397	KX298888
	Angariidae	*Angaria neglecta*	19,470	NC_028707
		*Angaria delphinus*	19,554	NC_031860
	Haliotidae	*Haliotis ovina*	16,531	NC_056350
		*Haliotis tuberculata*	16,521	FJ599667
		*Haliotis laevigata*	16,545	NC_024562
	Trochidae	*Stomatella planulata*	17,151	NC_031861
		*Gibbula umbilicalis*	16,277	NC_035682
		*Umbonium thomasi*	15,998	MH729882
		*Monodonta labio*	16,440	MK240320
	Turbinidae	*Bolma rugosa*	17,432	NC_029366
		*Lunella granulate*	17,190	NC_031857
		*Lunella correensis*	17,308	MN604179
	Tegulidae	*Tegula lividomaculata*	17,375	NC_029367
		*Tegula brunnea*	17,690	NC_016954
		*Chlorostoma argyrostomum*	17,780	KX298892
		*Omphalius rusticus*	18,067	NC_056356
		*Omphalius nigerrimus*	17,755	KX298895

**Table 2:** Annotation of the *Cellana toreuma* mitochondrial genome.

Gene	Strand	Location		Length	Codons	Intergenic Nucleotide*(bp)	Anticodon
		Start	Stop				
*cox1*	+	1	1542	1542	ATG/TAA	74	
*cox2*	+	1617	2315	699	ATG/TAA	21	
*trnD*	+	2337	2403	67		58	GTC
*atp8*	+	2462	2650	189	ATG/TAA	248	
*atp6*	+	2899	3393	495	ATG/TAA	34	
*trnT*	+	3428	3496	69		53	TGT
*nad5*	-	3550	5232	1683	ATG/TAA	39	
*trnH*	-	5272	5339	68		30	GTG
*trnQ*	-	5370	5438	69		24	TTG
*nad4*	-	5463	6815	1353	ATA/TAA	5	
*nad4l*	-	6821	7087	267	ATG/TAA	49	
*trnS2*	-	7137	7204	68		17	
*cob*	-	7222	8367	1146	ATG/TAG	20	
*nad6*	-	8388	8870	483	ATT/TAA	24	
*trnP*	-	8895	8962	68		−47	TGG
*nad1*	-	8916	9881	966	ATT/TAA	21	
*trnL2*	-	9903	9968	66		4	
*trnL1*	-	9973	10,041	69		−38	
*rrnL*	-	10,004	11,269	1266		87	
*trnV*	-	11,357	11,423	67		0	TAC
*rrnS*	-	11,424	12,310	887		0	
*trnY*	-	12,311	12,376	66		15	GTA
*trnM*	-	12,392	12,458	67		−2	CAT
*trnF*	-	12,457	12,524	68		2	GAA
*trnW*	-	12,527	12,595	69		8	TCA
*trnC*	-	12,604	12,670	67		643	GCA
*trnG*	+	13,314	13,380	67		7	TCC
*trnE*	+	13,388	13,455	68		0	TTC
*cox3*	+	13,456	14,235	780	ATG/TAG	22	
*trnR*	+	14,258	14,324	67		1	TCG
*trnN*	+	14,326	14,395	70		76	GTT
*nad3*	+	14,472	14,825	354	ATG/TAA	7	
*trnA*	+	14,833	14,900	68		0	TGC
*trnK*	+	14,901	14,973	73		13	TTT
*trnI*	+	14,987	15,054	68		31	GAT
*trnS1*	+	15,086	15,153	68		3	GCT
*nad2*	+	15,157	16,254	1098	ATA/TAG	5	

Intergenic nucleotide*(bp): positive values indicated the interval sequence of adjacent genes, and negative values indicated the overlapping of adjacent genes.

**Table 3:** Nucleotide compositions of the mitogenomes from five species in family Nacellidae of the Patellogastropoda.

	Lengh (bp)	A (%)	T (%)	G (%)	C (%)	A+T (%)	AT-Skew	GC-Skew
*Cellana toreuma*	16,260	28.9	39.5	19.9	11.7	68.4	−0.155	0.261
tRNAs	1497	34.8	34.8	16.8	13.6	69.6	0.000	0.103
rRNAs	2153	41.7	29.7	14.5	14.1	71.4	0.169	0.016
PCGs	11,133	26.5	39.8	17.3	16.4	66.3	−0.201	0.027
1st	5420	30.9	36.4	21.6	11.1	67.3	−0.083	0.319
2nd	5420	28.8	38.1	19.9	13.2	66.9	−0.140	0.201
3rd	5420	27.0	43.9	18.4	10.7	70.9	−0.238	0.264
*Cellana nigrolineata*	16,153	26.5	38.0	22.7	12.7	64.6	−0.179	0.283
tRNAs	1498	33.4	33.9	18.0	14.6	67.4	−0.007	0.104
rRNAs	2143	41.6	27.7	14.6	16.1	69.3	0.201	−0.047
PCGs	11,046	25.4	36.8	18.9	18.8	62.2	−0.184	0.003
1st	5385	28.1	36.3	23.5	12.2	64.3	−0.128	0.317
2nd	5384	26.0	37.9	22.9	13.2	63.9	−0.186	0.268
3rd	5384	25.5	40.0	21.9	12.7	65.4	−0.221	0.265
*Nacella clypeater*	16,742	27.5	38.6	19.9	13.9	66.1	−0.169	0.177
tRNAs	1560	32.6	33.7	19.0	14.7	66.3	−0.015	0.125
rRNAs	2222	43.1	27.5	14.6	14.8	70.6	0.221	−0.006
PCGs	11,283	26.3	38.9	17.6	17.2	65.2	−0.193	0.009
1st	5581	25.8	40.4	19.2	14.7	66.1	−0.221	0.132
2nd	5581	28.2	38.3	19.8	13.7	66.5	−0.151	0.182
3rd	5580	28.4	37.3	20.9	13.4	65.7	−0.134	0.217
*Nacella concinna*	16,761	27.1	38.9	20.4	13.6	66.0	−0.180	0.197
tRNAs	1501	32.6	34.1	18.7	14.6	66.8	−0.022	0.122
rRNAs	2216	44.0	27.1	14.2	14.7	71.1	0.237	−0.017
PCGs	11,286	25.7	38.7	18.1	17.5	64.4	−0.201	0.015
1st	5587	28.2	36.4	21.3	14.1	64.6	−0.127	0.202
2nd	5587	26.5	39.5	19.9	14.1	66.0	−0.198	0.171
3rd	5587	26.6	40.8	19.9	12.7	67.4	−0.212	0.220
*Nacella magellanica*	16,663	27.4	38.9	20.1	13.6	66.2	−0.174	0.192
tRNAs	1500	32.4	34.1	18.8	14.7	66.5	−0.026	0.124
rRNAs	2214	43.5	27.4	14.4	14.6	71.0	0.227	−0.008
PCGs	11,280	26.2	39.0	17.5	17.2	65.2	−0.197	0.009
1st	5555	28.0	37.0	21.5	13.5	65.0	−0.139	0.230
2nd	5554	27.7	38.0	20.1	14.2	65.7	−0.158	0.170
3rd	5554	26.4	41.6	18.8	13.2	68.1	−0.224	0.175

## Data Availability

Not applicable.
